# Genome-Wide Analysis Elucidates the Roles of *GhTIR1*/*AFB* Genes Reveals the Function of *Gh_D08G0763* (*GhTIR1*) in Cold Stress in *G. hirsutum*

**DOI:** 10.3390/plants13081152

**Published:** 2024-04-20

**Authors:** Xianliang Zhang, Cuicui Wu, Yutao Guo, Xiang Ren, Yongming Meng, Qi Gao, Fei Zhang, Yaping Wang, Jinggong Guo

**Affiliations:** 1 State Key Laboratory of Cotton Biology, Institute of Cotton Research, Chinese Academy of Agricultural Sciences, Anyang 455000, China; zhangxianliang@caas.cn (X.Z.); renxiang@caas.cn (X.R.); mengyongming2022@163.com (Y.M.); zmsqigao@163.com (Q.G.); zhangfei@caas.cn (F.Z.); 2 Western Research Institute, Chinese Academy of Agricultural Sciences (CAAS), Changji 831100, China; 3 Institute of Cotton Research, Shanxi Agricultural University, Yuncheng 044000, China; wucuicui19821021@126.com; 4 Institute of Plant Stress Biology, State Key Laboratory of Cotton Biology, Henan University, Kaifeng 475000, China; yutao_guo@163.com; 5 Sanya Institute of Henan University, Sanya 572025, China; yapingwang23@163.com

**Keywords:** *G. hirsutum*, *TIR1*/*AFBs*, cold stress

## Abstract

This study identified 13 *GhTIR1*/*AFB* members in *G. hirsutum* through bioinformatics methods and divided them into three subgroups by phylogenetic tree analysis. Motif and gene structure analysis showed that the genes in this family were highly conserved. Promoter *cis*-acting element analysis found that the promoters of *GhTIR1*/*AFBs* contained a large number of *cis*-acting elements in response to growth and development and abiotic stress. Further RT-qPCR results showed that *GhTIR1*/*AFB* genes responded to various abiotic stresses such as IAA, ABA, cold, and heat, and the expression levels of each gene changed obviously, especially *Gh_D08G0763* (*GhTIR1*), which responded significantly to cold injury. Using VIGS (virus-induced gene silencing) technology to silence *Gh_D08G0763* in the cold-tolerant cotton variety *ZM36*, it was found that the resistance of *ZM36* to cold damage was significantly reduced. The physiological response mechanism of the *Gh_D08G0763* in resisting cold damage was further analyzed through trypan blue staining of leaves and determination of enzyme activity levels. This study provided effective genetic resources for cotton cold-tolerance breeding.

## 1. Introduction

Auxin is one of the main hormones regulating plant growth. There are four auxin receptors: ABP1 (auxin binding protein 1) and its interacting transmembrane kinase TMK1-TMK4; TIR1 (transport inhibitor response 1) and its homologous AFB1–AFB5; SKP2a (S-phase kinase-associated protein 2a); and ARF3 (auxin response factor 3), also known as ETTIN or ETT [1].

Among auxin receptors, the TIR1/AFB (transport inhibitor response1/auxin signaling f-box) receptor is the most widely studied, and *TIR1*/*AFB*-mediated auxin signal transduction is the most widely studied and best characterized auxin signal pathway. In this pathway, TIR1/AFB binds with SKP1 to form a SCF^TIR1/AFB^ complex to function. The *TIR1*/*AFBs*-mediated auxin signal transduction pathway is affected by auxin concentration and is a typical auxin concentration sensing mechanism [2]. At low auxin concentrations, Aux/IAA recruits TOPLESS (TPL)/TPL-related protein (TPRs) and histone deacetylase (HDACs) to bind to ARF, preventing ARF from binding to downstream genes and thus inhibiting the expression of downstream genes. At high concentrations, IAA binds to SCF^TIR1/AFB^ to enhance its binding to Aux/IAA and activates ubiquitin ligase to ubiquitinate Aux/IAA, which is then degraded by 26S protease, thereby relieving the inhibition of transcription factor ARF [1,3].

*TIR1*/*AFBs* play an important role in plant development by regulating auxin signals, such as root growth, root cell wall, root tropism, stem development, leaf, flower, etc. For example, overexpression of *OsAFB6* in rice delays heading and increases cytokinin (CK) by inhibiting the expression of Gn1a; while decreasing the concentration of IAA in young panicles, overexpression of *OsAFB6* promotes inflorescence meristem development, resulting in large panicles, more grains per panicle, more primary branches, and increased grain yield [4]. Overexpression of *PtrFBL1* in poplar can promote stem growth [5]. Knockout of the *GmTIR1* and *GmAFB3* genes could reduce the number of nodules in soybeans [6].

In addition, the *TIR1*/*AFB*-mediated auxin signaling pathway is also involved in plant tolerance to abiotic or biotic stress [7,8]. In *Arabidopsis thaliana*, ABA treatment usually inhibits the production of lateral roots, but overexpression of *AtAFB2* has been proven to counteract this defect, indicating that abiotic stress can induce *TIR1*/*AFB* to a certain extent. In cucumber *(Cucumis sativus* L.), knockout of *CsTIR*/*AFB* can reduce the salt tolerance of *Arabidopsis thaliana*, while overexpression of *CsTIR*/*AFB* enhances the salt tolerance of transgenic *Arabidopsis thaliana*, indicating that *CsTIR*/*AFB* participates in auxin signal regulation, triggers auxin-mediated stress adaptation, and enhances salt stress tolerance through osmotic regulation [9]. *TIR1*/*AFB* family members have been found in different plant species, and some of them are reported to be involved in several different stress responses, including salt, cold, drought, and pathogen immunity [6,10].

Cold is one of the main abiotic stresses faced by plants, which limits plant growth, development, and seasonal distribution. When plants encounter low temperatures, their cold tolerance and freezing resistance are improved; that is, this adaptation process is called cold adaptation [11]. A cold injury can destroy the integrity and fluidity of the cell membrane and change its composition [12]. The higher the unsaturation of the plant cell membrane, the stronger the ability to cope with cold stress, which can be used as one of the important indicators to evaluate the response of plants to low temperature stress [13]. When plants are subjected to low-temperature stress, it can induce the active expression of some enzymes in the body to eliminate the accumulation of reactive oxygen species (ROS) and prevent plant damage. Among them, antioxidant enzymes mainly include superoxide dismutase (SOD), peroxidase (POD), catalase (CAT), ascorbate peroxidase (APX), and so on [14]. 

Many plants, such as *Arabidopsis thaliana* and oilseed rape, have evolved a series of complex cold adaptation mechanisms involving extensive physiological, biochemical, and metabolic changes [15,16,17]. Cotton, which originated in tropical and subtropical regions, is a temperature-loving crop, and the optimum temperature for growth and development is about 22–30 °C. The temperature below 15 °C is a low-temperature stress for cotton, which will have a certain impact on the growth, development, and production of cotton [11,18]. Therefore, how to improve the environmental adaptability of cotton and optimize the quality of cotton varieties is an important research direction. 

At present, *TIR1*/*AFB* genes in many plants have been identified and functionally verified, but there are few studies on cotton auxin receptor *TIR1*/*AFBs* [19,20], and their response to abiotic stress has not been reported. Therefore, in this study, we identified *TIR1*/*AFBs* in cotton and studied the role of *TIR1*/*AFB* genes in the mechanism of cold resistance in cotton, so as to provide more insights into the mechanism of cotton responding to cold stress. 

## 2. Material and Methods

### 2.1. Identification of TIR1/AFBs in G. hirsutum and Phylogenetic Tree Construction

Using the *TIR1* sequence in *Arabidopsis* as a reference, the *G. hirsutum* genome database (NAU-NBI_v1.1) was queried from the CottonFGD website (https://cottonfgd.org (accessed on)), and the *GhTIR1*/*AFB* genes were identified by BLASTP. The TIR1/AFB protein domains were analyzed using the Hidden Markov Model (HMM) from the Pfam database (http://pfam.xfam.org/ (accessed on 21 June 2023)). The TIR1 domains were confirmed by Pfam accession numbers PF18791 and PF18511. The E-value threshold for the HMMER search was set at 1 × 10^−10^ to obtain possible proteins. 

Multiple alignments of all the predicted GhTIR1/AFB and AtTIR1 protein sequences were performed using ClustalX 2.0 [21]. An unrooted phylogenetic tree was generated using the neighbor-joining (NJ) method and the amino acid p-distance model in MEGA 11.0 [22]. A total of 1000 bootstrap replicates were used to assess the reliability of interior branches.

### 2.2. Analysis of Motif and the Exon/Intron Structure of GhTIR1/AFB Genes

The online program of MEME (http://meme-suite.org/ (accessed on 21 June 2023)) [23] was employed to determine the conserved motifs of *GhTIR1*/*AFBs* with the following optimum parameters: a motif width of 6–50 amino acids and a maximum of 10 motifs. The identified motifs were annotated using the program InterProScan [24]. The exon/intron structures of *GhTIR1*/*AFBs* were retrieved according to the GFF annotation file information of *G. hirsutum* using the gene structure display server (GSDS) program (http://gsds.cbi.pku.edu.cn/ (accessed on 21 June 2023)) [25]. 

### 2.3. Analysis of Cis-Elements of Upstream Sequences

To determine the *cis*-elements of the predicted promoters, the 2000 bp genomic DNA sequences upstream of the initiation codon (ATG) of all *GhTIR1*/*AFBs* were employed to search the PLACE database (http://www.dna.affrc.go.jp/PLACE/signalscan.html (accessed on 21 June 2023)) [26]. The prediction results were visualized using TBtools software (TBtools-II_v2.082) [27].

### 2.4. Transcriptome Analysis

The RNA-seq data (accession: PRJNA248163) of abiotic stress (4 °C cold, 37 °C hot, 200 mM salt, and 200 g/LPEG) at different time laps (0, 1, 3, 6, and 12 h for each treatment) were downloaded from the NCBI database (https://www.ncbi.nlm.nih.gov/ (accessed on 21 June 2023)). All gene expression levels were normalized by log_2_ (FPKM + 1). The heat maps were generated through TBtools software [27].

### 2.5. Plant Materials and Treatments

*G. hirsutum* cultivar ZM36 was grown in a climate-controlled green house (light/dark cycle: 16 h at 28 °C/8 h at 22 °C) and was treated with 100 μM IAA, 300 μM ABA, 37 °C, 12 °C, and 30% PEG6000 (root materials of two-week seedlings were taken at 1, 6, 12, and 24 h after treatment, and the material at 0 h was used as a control). All samples were immediately frozen in liquid nitrogen and stored at −80 °C. Three biological replicates were performed for each sample. Tobacco (Nicotiana benthamiana) was cultivated under 16 h light (25 °C)/8 h dark (22 °C) conditions in the Cotton Research Institute of the Chinese Academy of Agricultural Sciences (Anyang, China). 

### 2.6. Subcellular Localization Analysis

The full-length coding sequence of Gh_D08G0763 was amplified and cloned into the pBI1300-GFP vector driven by the constitutive Cauliflower mosaic virus 35S promoter. The fusion vector was transformed into *Agrobacterium tumefaciens* strain GV3101, and then injected into *Nicotiana benthamiana* leaves for 48–72 h by *Agrobacterium*-mediated transient transformation [28]. The GFP fluorescence in the leaves was observed by using confocal microscopy (Leica, SP8, Heidelberg, Germany).

### 2.7. Yeast Two-Hybrid Assay

The coding sequences of Gh_D08G0763, Gh_A07G2125, Gh_A10G0207, Gh_D10G0187, and Gh_D11G0671 were amplified with the primers listed in Table 1. All PCR products were cloned into pGBKT7. The coding sequence of GhSKP1 was cloned into the pGADT7 vector. The yeast two-hybrid assay was performed using the Match Maker GAL4 Two-hybrid System 2 (Clontech, Mountain View, CA, USA). The assays were performed as described previously [29].

### 2.8. VIGS for Gene Functional Verification

The VIGS assay was performed as previously described [30]. The conserved fragments of Gh_D08G0763 and GhCLA1 (used as a positive control) were amplified using the primers listed in Table 1. The amplified fragments were subsequently cloned into the pTRV2 (pYY13) vector to construct pTRV2-Gh_D08G0763 and pTRV2-GhCLA1. Then, these constructs were transformed into Agrobacterium tumefaciens GV3101 [31]. Then, the Agrobacterium cultures were injected into cotyledons of two-week-old cotton seedlings (ZM36). Next, expression of Gh_D08G0763 was detected by RT-qPCR two weeks after injection. Control and Gh_D08G0763-silenced cotton plants were placed in an incubator at 12 °C for 24 h [30]. 

### 2.9. Trypan Blue Staining Experiment

Cotton leaf experimental materials and control materials from VIGS plants were boiled for 5–8 min, stained with trypan blue solution diluted to 0.4% PBS for 6–8 h at room temperature, and then decolorized with 95% ethanol to observe the distribution of leaf tissue damage.

### 2.10. Determination of Enzyme Activity Level of Gene Expression

When the seedlings grew to two leaves and one heart, the VIGS injection phenotype emerged, and cold treatment was carried out for 24 h. The experimental materials and control materials of stressed cotton leaves from VIGS plants were selected. The activities of superoxide dismutase (SOD), peroxidase (POD), and catalase (CAT) were determined by the peroxidase assay kit, the peroxidase assay kit, and the catalase assay kit (Suzhou Grace Biotechnology Co., Ltd., Suzhou, China), respectively. The absorbance values were observed at 560 nm, 470 nm, and 510 nm, respectively.

### 2.11. RNA Extraction and RT-qPCR Analysis

The total RNA of the collected samples was extracted using the RNAprep Pure Plant kit (Tiangen, Beijing, China) according to the manufacturer’s instructions. First-strand cDNA was synthesized via reverse transcription using the PrimeScript RT Reagent Kit (Takara, Shiga, Japan). Primer 5.0 software was used to design the gene-specific primers for RT-qPCR (Table 1). GhUBQ7 (GenBank: DQ116441) was used as an internal reference control. The RT-qPCR experiments were performed using SYBR Premix Ex Taq (Takara) on an ABI 7500 real-time PCR system (Applied Biosystems, Waltham, MA, USA) with three replicates. The details of the protocol were as follows: (Step 1) initial denaturation step of 30 s at 95 °C; (Step 2) 40 cycles of 5 s at 95 °C, and 34 s at 60 °C; and (Step 3) melting curve analysis. The 2^−ΔΔCT^ method was used to calculate the relative expression levels of GhTIR1/AFBs [32]. *T*-tests were employed for statistical analyses.

### 2.12. Primer Sequence Design 

The gene sequences were obtained from the NCBI database (https://www.ncbi.nlm.nih.gov/ (accessed on 21 June 2023)) and CottonFGD (https://cottonfgd.net/ (accessed on 21 June 2023)). The primers were designed through Premier 5.0 software. The primers were synthesized, and subsequent sequencing was completed by Beijing Qingke Biotechnology Co., Ltd. (Beijing, China). The primer sequences are shown in Table 1. 

## 3. Results and Analysis

### 3.1. Identification and Evolutionary Analysis of TIR1/AFBs in G. hirsutum

**Figure 1:**
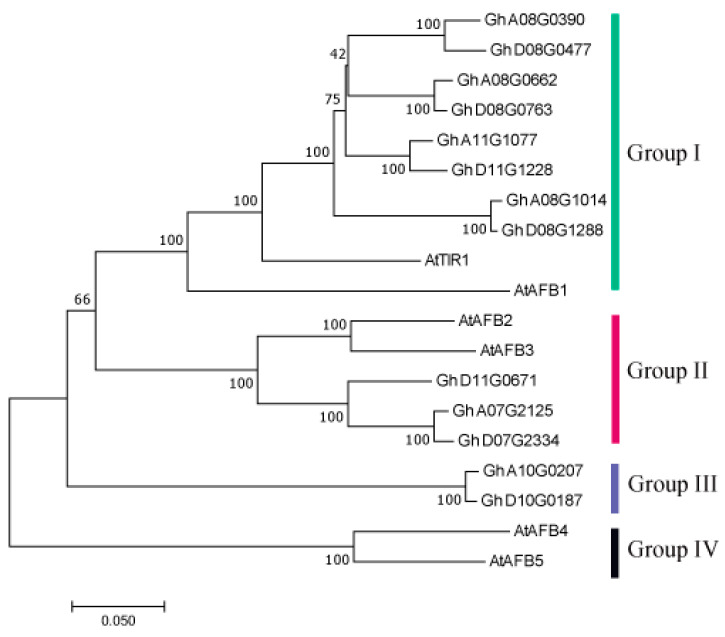
Phylogenetic tree of *TIR1*/*AFB* proteins in *G. hirsutum* and *Arabidopsis thaliana*.

Based on the *TIR1* sequence of *Arabidopsis thaliana*, 13 genes were found in the *G. hirsutum* genome database (NAU-NBI_v1.1) by blast. After comparing the results from the CDD and SMART, a total of 13 genes were finally identified as belonging to the *GhTIR1*/*AFB* gene family. To further analyze the evolutionary relationship of the *TIR1*/*AFB* gene family in *G. hirsutum* and *Arabidopsis thaliana*, a phylogenetic tree containing 13 *GhTIR1*/*AFB* genes and 6 *AtTIR1* genes was constructed (Figure 1). This tree was phylogenetically divided into four groups. Group I contained *Gh_A08G0390*, *Gh_D08G0477*, *Gh_A08G0662*, *Gh_D08G0763*, *Gh_A11G1077*, *Gh_D11G1228*, *Gh_A08G1014*, *Gh_D08G1288*, *AtTIR1*, and *AtAFB1*. This indicated the above eight genes in Group I of *G. hirsutum* were highly homologous to the *Arabidopsis TIR1* and *AFB1*, which functioned as auxin receptors. Group II included *Gh_D11G0671*, *Gh_A07G2125*, *Gh_D07G2334*, *AtAFB2*, and *AtAFB3*. And Group III just included *Gh_A10G0207* and *Gh_D10G0187*; there were no Arabidopsis *TIR1*/*AFB* genes in this group. In Group VI, there were only two Arabidopsis *TIR1*/*AFB* genes, *AtAFB4* and *AtAFB5*. The results of phylogenetic tree analysis showed that some members of the *TIR1*/*AFB* gene family had species specificity.

### 3.2. Analysis of Conserved Protein Motifs and Gene Structure of GhTIR1/AFBs

**Figure 2:**
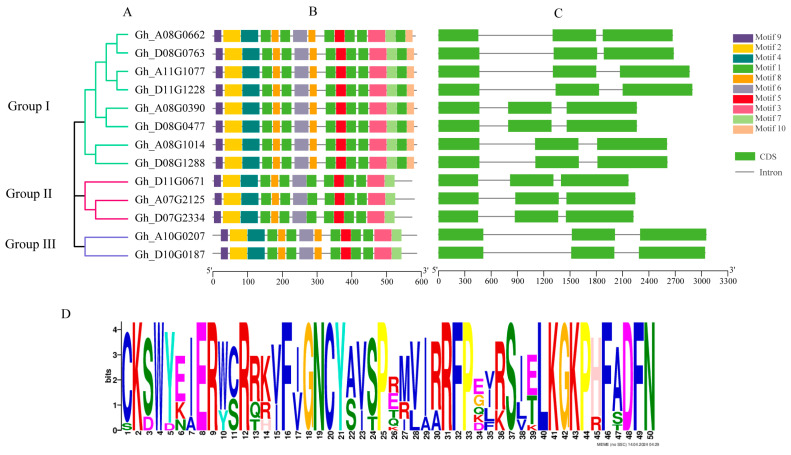
Phylogenetic relationships, motif analysis, and gene structure of *GhTIR1*/*AFBs*. (**A**). Phylogenetic analysis of GhTIR1/AFB proteins using MEGA 11 via the neighbor-joining (NJ) method with 1000 bootstrap replicates. (**B**). Ten motifs of the GhTIR1/AFB proteins were determined using MEME. Different colors represent different motifs. (**C**). Exon–intron organization of the *GhTIR1*/*AFBs* family. The exons and introns are indicated with green-filled boxes and gray lines, respectively. (**D**). Sequence logo of the partial conserved domain (F-BOX).

To further understand the conservation and diversification of the *GhTIR1*/*AFBs*, the conserved motifs and exon–intron structures were investigated and shown in Figure 2. We identified 10 conserved motifs of the GhTIR1/AFB proteins using MEME (Figure 2B). Group I contained all 10 types of motif and especially had 6 motif1. Group II and Group III both had nine types of motif except motif 10. The motif types and numbers in each group were consistent. 

In addition, all *GhTIR1*/*AFB* family genes contain three exons and two introns, and the distribution pattern was relatively consistent, especially among homologous gene pairs. The results indicated that there was a high degree of conservation among different genes (Figure 2C). All proteins contained the conserved domain F-BOX (Figure 2D).

### 3.3. Analysis of Cis-Acting Elements in the Promoter Regions of GhTIR1/AFBs

**Figure 3:**
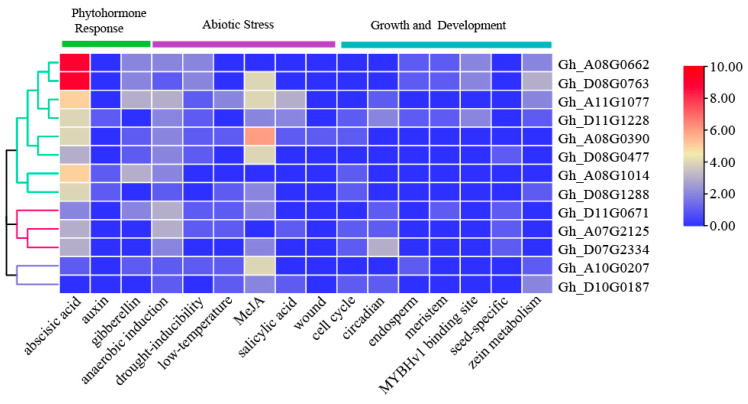
Distribution of *GhTIR1*/*AFBs* promoter *cis*-acting elements in *G. hirsutum*. The evolutionary tree is on the left, and gene names are on the right. The upper part is the type of *cis*-acting component, and the bottom coordinate is the function of the cis-acting component. The color in the figure indicates the number of the cis-acting element *GhTIR1*/*AFBs* in the 2000 bp sequence upstream of the start codon, ranging from 0 to 10.

The analysis of promoter *cis*-elements was an important research method to understand gene transcription and expression regulation. In order to explore the regulatory mechanism of the *GhTIR1*/*AFB* genes in *G. hirsutum*, the 2000 bp upstream sequences of 13 *GhTIR1*/*AFB* genes were obtained from the cotton database, and *cis*-elements were identified by PlantCARE (https://bioinformatics.psb.ugent.be/webtools/plantcare/html/ (accessed on 21 June 2023)), which is divided into three types: plant growth and development, phytohormones, and abiotic stress and displayed the number of *cis*-elements on the promoter of *GhTIR1*/*AFBs* in *G. hirsutum* in the form of a heat map (Figure 3).

In plant growth and development aspects, there were meristem expression, circadian control, cell cycle regulation, seed-specific regulation, and other types present in the promoter region of *GhTIR1*/*AFBs*.

The phytohormone response included abscisic acid, auxin, and gibberellin, among which abscisic acid was the most attractive, and its conserved sequence was PYCGTGGC. It can be seen from the heat map that, except for *Gh_D10G0187*, all other *GhTIR1*/*AFB* genes contain abscisic acid elements. Among them, *Gh_A08G0662* and *Gh_D08G0763* had far more abscisic acid elements than other genes, indicating that the functions of these two genes might be regulated by abscisic acid. 

Additionally, a large number of cis-acting elements related to abiotic stress appeared in the promoter region of the *GhTIR*/*AFB* genes, such as anaerobic induction, drought, low temperature, MeJA, et al. Most genes had cis-elements related to MeJA, and *Gh_A08G0390* had the most cis-elements related to MeJA. The LTR *cis*-acting element was an element related to cold stress. Cold damage was one of the major environmental factors that affected the growth and production of cotton. It was also a focus of attention in cotton research.

### 3.4. Expression Analysis of GhTIR1/AFBs under Phytohormone and Abiotic Stress

According to the analysis results of cis-acting elements, *GhTIR1*/*AFBs* were largely related to the response to phytohormones and abiotic stress. In this study, the published transcriptome data of TM-1 under four abiotic stresses (cold, heat, salt, and drought) were used for heat map analysis (Figure S1). The results showed that, except for *GhA08G0390* and *GhD08G0477*, the other genes in the GhTIR1/AFB family responded to various abiotic stresses. We further identified some of the GhTIR1/AFB family genes in the cold-tolerant material ZM36 by RT-qPCR under various hormones and abiotic stresses.

**Figure 4:**
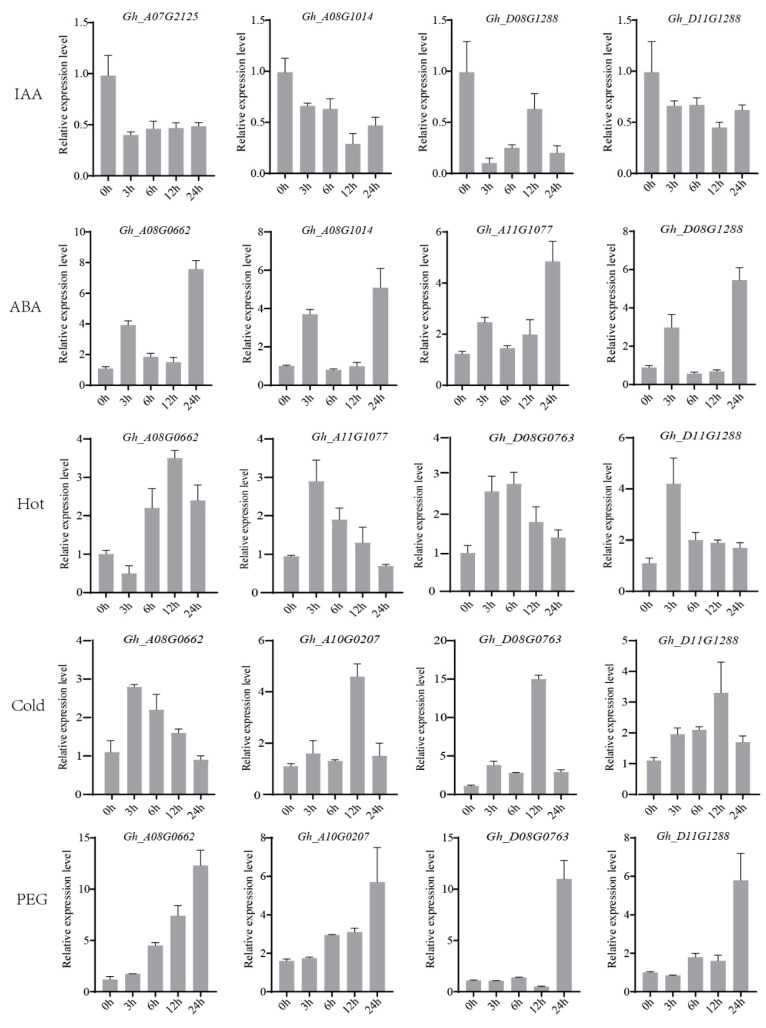
Expression patterns of the *GhTIR1*/*AFB* genes under IAA (100 μM), ABA (300 μM), heat (37 °C), cold (12 °C), and drought (30% PEG6000) at different time laps (3, 6, 12, and 24 h) shown as are shown as relative expressive. Error bars indicate standard deviations among three independent biological replications (n = 3). *GhUBQ7* was used as the internal control.

*TIR1*/*AFBs* acted as auxin receptors and were induced by IAA to produce a series of responses [33]. Whether the *GhTIR1*/*AFBs* identified in *G. hirsutum* responded to IAA induction was a focus of our attention. The RT-qPCR results showed that IAA had an inhibitory effect on *Gh_A07G2125*, *Gh_A08G1014*, *Gh_D08G1288*, and *Gh_D11G1288*, and *Gh_D08G1288* showed the most obvious inhibitory effect (Figure 4). And *Gh_A08G0662, Gh_A08G1014*, *Gh_A10G1077*, and *Gh_D08G1288* were induced by ABA, and the induction effect was significant at 3 h and 24 h. However, the expression levels of *Gh_A08G1014* and *Gh_D08G1288* decreased at 6 h, which might be related to plant feedback regulation.

Additionally, the RT-qPCR results of heat stress (37 °C) showed that the expression of *Gh_A08G0662* decreased at 3 h after treatment, while the expression of other genes increased at this time point. *Gh_A11G1077* and *Gh_D11G1288* reached the highest at 3 h, and the expression gradually decreased. *Gh_A08G0662* and *Gh_D08G0763* peaked at 12 h and 6 h, respectively. 

When treated with cold stress at 12 °C, the expression levels of the *GhTIR1*/*AFBs* increased after induction. Among them, the expression level of *Gh_D08G0763* increased more than ten times at 12 h, so it could be speculated that this gene played a certain role in cotton resistance to cold stress. 

And the RT-qPCR results of 30% PEG6000 showed that the expression levels of the selected *GhTIR1*/*AFBs* were gradually increased after treatment. The above results provided valuable information for further research on the role of *GhTIR1*/*AFBs*.

### 3.5. Subcellular Localization of Gh_D08G076

**Figure 5:**
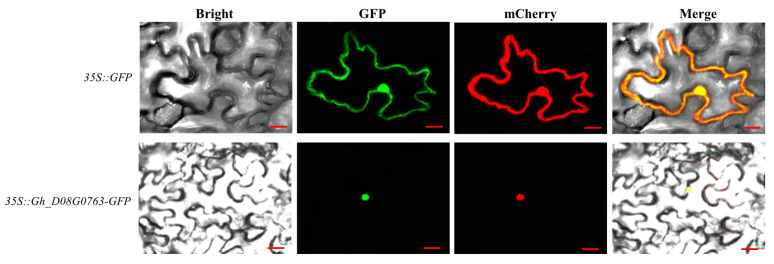
Subcellular localization of *Gh_D08G0763*. The nucleus labeling by mCherry was shown as red fluorescence. GFP driven by the CaMV35S promoter was used as a control. Fluorescence was observed 48–72 h post-Agrobacterium infiltration using confocal microscopy. Bar = 50 μm.

According to previous results, the expression level of *Gh_D08G0763* increased significantly when cotton was treated at a low temperature. Therefore, this experiment wanted to explore whether this gene had a function under cold stress. First, we cloned the CDS sequence of *Gh_D08G0763* into the p35S:S1300-GFP vector and transformed the constructed plasmid into tobacco leaves for observation. As a result, fluorescent signals were detected in the nuclear region of epidermal cells, indicating that the gene functions in the nucleus (Figure 5). 

### 3.6. Yeast Two-Hybrid Verification of the Interaction between GhTIR1/AFBs and GhSKP1 Protein

**Figure 6:**
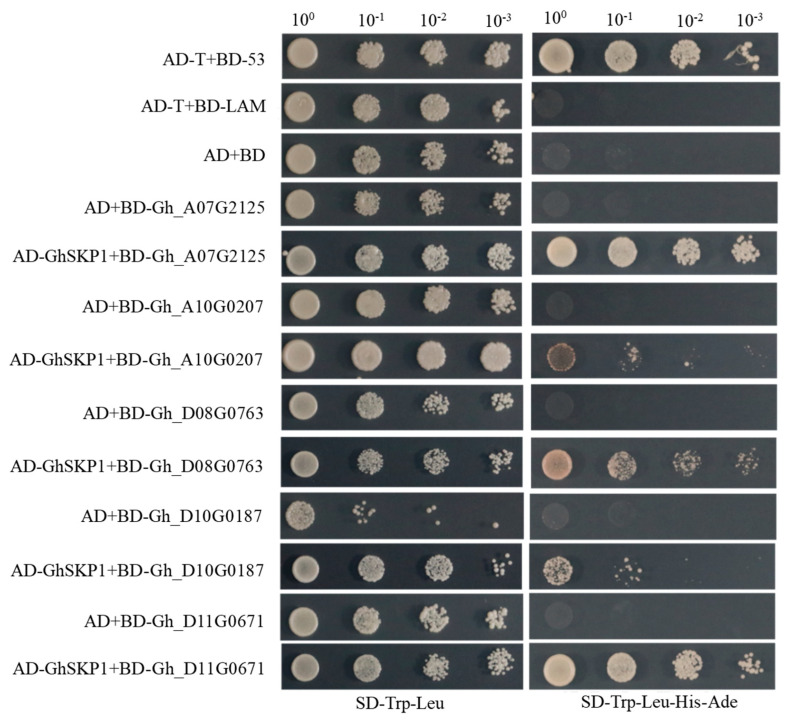
Yeast-two-hybrid-verified interaction between GhTIR1/AFBs and GhSKP1 protein. BD-53 and AD-T were used as positive controls, BD-LAM and AD-T were used as negative controls, and BD and AD were used as blank controls. BD: pGBKT7 vector; AD: pGADT7 vector; AD-T: pGADT7-T; BD-53: pGBKT7-53; BD-LAM: pGBKT7-Lam.

GhTIR1/AFBs combined with SKP1 to form the SCF^TIR1/AFB^ complex in the auxin signaling pathway and played important functions in downstream [29]. The CDS sequences of *Gh_D08G0763*, *Gh_A07G2125*, *Gh_D10G0187*, *Gh_A10G0207*, and *Gh_D11G0671* were cloned into pGBKT7, respectively, and *GhSKP1* was cloned into pGADT7 (Figure 6). On the basis of self-activation detection (Figure S2), yeast two-hybrid experiments were further carried out. The results were observed on non-selective SD-Trp-Leu agar plates and selective SD-Trp-Leu-His-Ade agar plates. As shown in Figure 6, the GhSKP1 protein could interact with GhTIR1/AFBs, respectively.

### 3.7. Phenotype Observation of Gh_D08G0763 Knock-Out by VIGS

**Figure 7:**
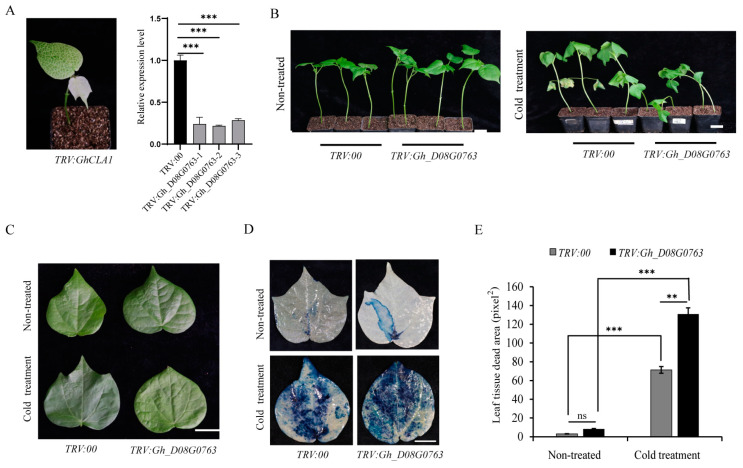
(**A**). *TRV:CLA1* appeared albino, and *Gh_D08G0763* was silenced efficiency detection. Samples were taken when the VIGS plant reached three true leaves, and the penultimate true leaf was taken, the same as (**C**,**D**). *GhUBQ7* was the internal reference gene in qRT-PCR, and a *T*-test was used to analyze three biological replicates of each sample. **: *p* < 0.01; ***: *p* < 0.001. (**B**). Plant phenotypic characteristics after *Gh_D08G0763* is silenced at the third true leaf of the cotton seedlings. Bar = 2 cm. (**C**). Leaf phenotypic characteristics after *Gh_D08G0763* silencing. Bar = 2 cm. (**D**). Trypan blue experiment. Bar = 2 cm. (**E**). Comparison of leaf tissue death area between *TRV:00* and *TRV:Gh_D08G0763* plants. ns: no significance.

ZM36 was a variety bred by the cotton research institute of the Chinese Agricultural Academy and had good cold resistance. Therefore, ZM36 conducted the VIGS experiment, which was employed to investigate the function of *Gh_D08G0763* in cotton plants [30,34,35]. To monitor VIGS efficiency, the *GhCLA1* gene was also silenced and used as a positive control (*TRV:GhCLA1*). Expression of *Gh_D08G0763* was detected after *TRV:GhCLA1* plants showed an albino phenotype (Figure 7A). The expression of *Gh_D08G0763* in *TRV: Gh_D08G0763* plants decreased by 55–70% compared with the control group. 

Subsequently, *TRV:00* and *TRV:Gh_D08G0763* plants were placed in an incubator at 12 °C for 24 h. After cold treatment, *TRV:00* plants showed obvious wilting, while *TRV: Gh_D08G0763* plants showed serious wilting in ZM36 (Figure 7B). Therefore, we speculated that *Gh_D08G0763* might play a positive regulatory role in cold stress in cotton. At the same time, we also observed the third true leaf of the cotton seedlings in the two groups of materials. As shown in Figure 7C, the leaves of *TRV: Gh_D08G0763* had more serious wilting than *TRV:00*, and the leaves of *TRV: Gh_D08G0763* turned yellow due to the low temperature. This showed that silent *Gh_D08G0763* could reduce the resistance to cold in cotton, which suggested *Gh_D08G0763* may play a positive regulatory role in cotton’s response to cold stress and may help cotton resist cold injury.

### 3.8. Trypan Blue Staining Analysis of VIGS Plant Leaves 

In order to further explore the response of different experimental materials to cold stress, we used trypan blue to dye the experimental materials. Trypan blue was an organic compound that was often used as a cell-reactive dye to detect the integrity of the cell membrane and whether cells survived or not. Living cells could not be stained blue, while dead cells could be stained light blue. Observing the distribution of leaf tissue damage results, *TRV: Gh_D08G0763* leaves had a larger blue area than *TRV:00*, indicating that the cells in the leaf died on a large scale due to low temperature damage (Figure 7D,E). The results demonstrated that *Gh_D08G0763* was a key gene for the cold stress response in cotton and could help cotton resist cold injury. 

### 3.9. Determination of Enzyme Activity in Plants Silenced by VIGS

**Figure 8:**
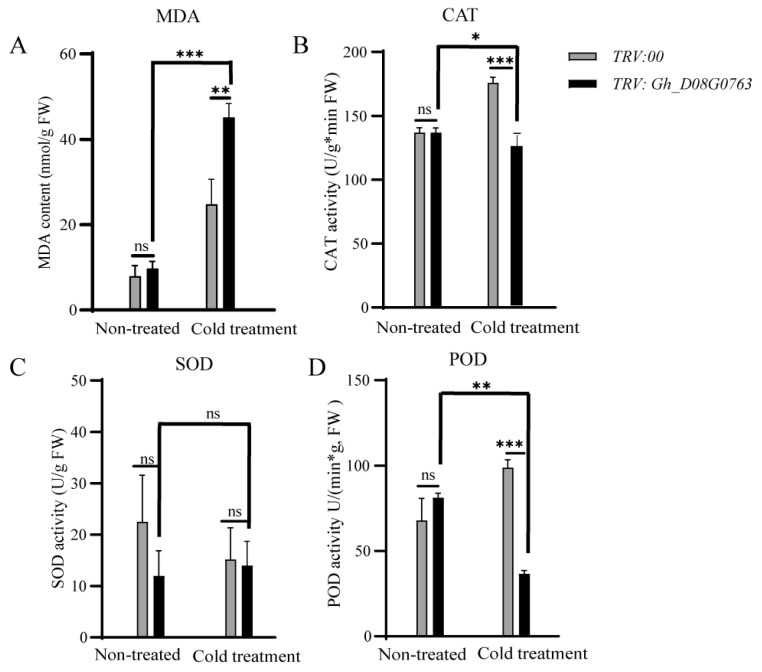
(**A**). Content detection of MDA between *TRV:00* and *TRV:Gh DO8G0763* lines. (**B**). Content detection of CAT between *TRV:00* and *TRV:Gh DO8G0763* lines. (**C**). Content detection of SOD between *TRV:00* and *TRV:Gh DO8G0763* lines. (**D**). Content detection of POD between *TRV:00* and *TRV:Gh DO8G0763* lines. The significance of the difference was analyzed with a two-tailed *t*-test. The error bars represent the mean ± SE. *: *p* < 0.05; **: *p* < 0.01; ***: *p* < 0.001; ns: no significance.

Malondialdehyde (MDA) content and catalase (CAT) activity were two important factors affecting plant cold tolerance [36]. The lower MDA content and the higher CAT activity indicated that the plant had stronger cold resistance [36]. In order to determine the difference in cold tolerance of *Gh_D08G0763* silenced plants, we measured the MDA content and CAT of the experimental materials. The results showed that the MDA content in leaves increased significantly and the CAT activity decreased significantly compared with the control (Figure 8), indicating that *Gh_D08G0763* affected the plant’s response to cold. In addition, the contents of SOD and POD could also reveal the response mechanism of cotton to cold stress. The results showed that the accumulation of POD was significantly inhibited in *Gh_D08G0763* silenced plants after cold treatment, but the SOD activity changed little. After cold treatment, the MDA index of *Gh_D08G0763* silenced plants increased significantly, while the CAT and SOD index decreased significantly (Figure 8). The above results suggested that *Gh_D08G0763* might be used as a functional gene to regulate cotton resistance to cold stress by promoting the expression of CAT and POD and inhibiting the production of MDA. 

## 4. Discussion

### 4.1. Bioinformatics Analysis of the GhTIR1/AFB Gene in G. hirsutum

*TIR1* was first isolated in 1997 and named as the transport inhibitor response protein [37]. The TIR1/AFB protein belonged to a subfamily of the F-box protein family. It had a F-box domain at the N-terminal and several leucine-rich repeat (LRR) domains at the C-terminal. Studies showed that the TIR1/AFB protein was a kind of nuclear protein that could interact with the SKP1 homologous protein (ASK1) to form the SCF protein complex. At present, six auxin receptor members have been found in *Arabidopsis thaliana*: *TIR1*, *AFB1*, *AFB2*, *AFB3*, *AFB4*, and *AFB5*. According to the phylogenetic tree, they could be divided into three categories: *TIR1*/*AFB1*, *AFB2*/*AFB3*, and *AFB4*/*AFB5* [1]. Among auxin receptors, *TIR1*/*AFB* was the most studied. *TIR1*/*AFBs* play an important role in plant development and stress by regulating the auxin signal.

In this study, 13 *TIR1*/*AFB* genes were found in *G. hirsutum*. The results of phylogenetic tree analysis of *G. hirsutum* and *Arabidopsis* were divided into four groups, of which eight genes were clustered with *TIR1* and *AFB1* in *Arabidopsis thaliana* (Figure 1). Group I might be a class of genes exercising auxin receptor function in *G. hirsutum* and an important direction of follow-up research. According to the reports on *AFB2* and *AFB3* in *Arabidopsis thaliana* [38], we speculated that three cotton genes in Group II are conserved in evolution and their functions might be similar to those in Group I. However, *GhA10G0207* and *GhD10G0187* in group III might be functionally different from other genes. There were only *AFB4* and *AFB5* in *Arabidopsis thaliana* in Group IV. According to the study [39,40], *AtAFB4* and *AtAFB5* were quite different from *AtTIR1* in terms of homology and gene structure. From the above grouping, it showed that some *TIR1*/*AFB* genes in cotton and *Arabidopsis* had their own species specificity. In addition, *GhTIR1*/*AFBs* were highly conservative, especially among genes in the same group, their motif and gene structure were almost the same, suggesting that different genes in the same group performed the same function (Figure 2).

### 4.2. The GhTIR1/AFB Gene Family Responded Positively to Abiotic Stress

As an auxin receptor, *AtTIR1*/*AFBs* were induced by IAA to produce a series of responses [41], and the auxin signal transduction pathway mediated by *TIR1*/*AFBs* was affected by auxin concentration, which was a typical auxin concentration sensing mechanism [2]. Whether the related family genes in *G. hirsutum* responded to the induction of IAA, the expression levels of some candidate genes were detected by RT-qPCR to apply 100 μM IAA. The results showed that IAA treatment inhibited the four *GhTIR1*/*AFBs* detected, which was consistent with the interaction between *TIR1s* and IAA; that is, when the concentration of auxin increased, *TIR1s* and Aux/IAA proteins were promoted by IAA. It could interact to form the SCF^TIR1/AFBs^ complex and then mediate the degradation of the Aux/IAA protein [11] through the ubiquitination of the 26S proteasome.

In addition, as one of the important plant hormones, ABA could not only promote seed germination and regulate the rapid response of plants to stress, but also interact with other plant hormones [42,43,44]. In this study, the results of *cis*-acting element analysis showed that most *GhTIR1*/*AFB* genes contained ABA-functional elements, and the results of qRT-PCR after exogenous ABA treatment also proved that multiple *GhTIR1*/*AFB* genes positively responded to ABA, indicating that *GhTIR1*/*AFBs* might be regulated by abscisic acid. The kind of regulatory mechanism between *GhTIR1*/*AFBs* and ABA was also worth studying. In addition, there were a large number of *cis* elements related to low temperature, drought, defense, and stress responsiveness, and other responses in the promoters of several *GhTIR1*/*AFBs* in *G. hirsutum* (Figure 3). These results showed that *GhTIR1*/*AFBs* were involved in the regulation of abiotic stress.

In this study, qRT-PCR results showed that multiple *GhTIR1*/*AFBs* responded to cold, heat, drought, and other stresses. Among them, *Gh_D08G0763* was especially prominent in cold stress treatment, and the expression level of *Gh_D08G0763* at 12 h was ten times higher than that before treatment (Figure 4), indicating that *Gh_D08G0763* was very likely to be involved in the regulation of cold stress.

### 4.3. Possible Regulatory Mechanisms of Gh_D08G0763 in Response to Cold Stress

In this study, subcellular localization results showed that *Gh_D08G0763* was localized in the nucleus, indicating that *Gh_D08G0763* functioned as a nuclear protein. Yeast two-hybrid results showed that GhTIR1/AFBs had an obvious interaction with GhSKP1, indicating that the complex produced by Gh_D08G0763 and GhSKP1 functioned in the nucleus.

VIGS technology was further used to silence the *Gh_D08G0763* in the cold-resistant material ZM36 (Figure 7A). After VIGS plants were cold-treated at 12 °C for 24 h, the VIGS material wilted more obviously than under normal conditions (Figure 7B). Furthermore, in the trypan blue staining analysis experiment, the leaves of the silenced material turned blue in the largest area (Figure 7D), indicating that the leaf cells of the silenced material died on a large scale after low temperature stress. Silencing *Gh_D08G0763* significantly reduced the cold resistance of ZM36. After cold treatment, the activities of POD, CAT, and SOD decreased in varying degrees, especially CAT and POD, which decreased significantly, while the MDA content increased significantly (Figure 8). The above results all indicated that *Gh_D08G0763* might be a key gene in cotton cold stress response and play a positive regulatory role under cold stress, and the gene might function by regulating the production of reactive oxygen species.

**Figure 9:**
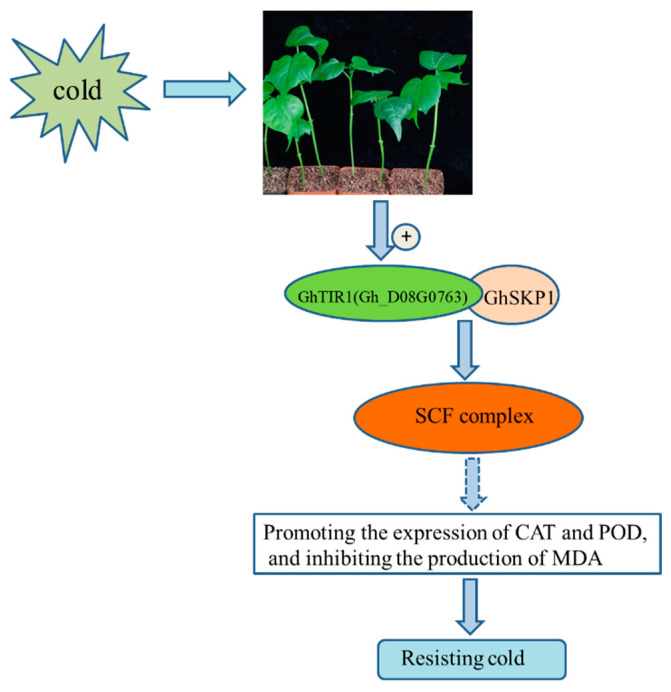
A schematic model of *Gh_D08G0763* is involved in the regulation of the cold stress response. Solid arrows represented published regulation models or validated results in this study, while dashed arrows represented putative regulatory pathways.

Combining the above phenotypic results after cold treatment, we inferred the regulatory pathway diagram of *Gh_D08G0763* during cold stress (Figure 9). That is, when cotton plants were subjected to cold stress, the expression of *Gh_D08G0763* in the cell nucleus increased, forming the SCF^Gh_D08G0763^ complex with *GhSKP1*. It was speculated that the cold stress response mediated by the auxin signaling pathway played a role in regulating the production of related reactive oxygen species in the plant. However, the related research needed to be further verified.

## 5. Conclusions

This study performed a genome-wide analysis of the phylogeny of the *TIR1*/*AFB* gene family in *G. hirsutum*. Promoter *cis*-acting element analysis showed that there were a large number of *cis*-acting elements responding to growth and development and abiotic stress in the promoters of *GhTIR1*/*AFBs*. RT-qPCR results suggested that *Gh_D08G0763* responded to hot, cold, and PEG, especially responding significantly under cold treatment. *Gh_D08G0763* was silenced through VIGS in ZM36, and the wilting level was more serious in silencing materials after 24 hours of cold treatment. Furthermore, trypan blue staining and enzyme activity experiments showed that silencing *Gh_D08G0763* could reduce cold resistance. These findings facilitate further investigations into the cold-tolerance breeding of *G. hirsutum*.

## Figures and Tables

**Table 1:** Primer sequences.

Prime Name	Prime Sequence (5′-3′)
Gh_D08G0763-GFP-F	TCTAGAAAGCTTCTGCAGATGCATAAGAAAATGGC
Gh_D08G0763-GFP-R	CTTGCTCACCATGGTACCGAAAGCCTCAATCCAGA
GhSKP1-AD-F	GAGGCCAGTGAATTCATGGCTTCTACGGGTCGGAA
GhSKP1-AD-R	GAGCTCGATGGATCCGCTCAAACGCCCATTGATTC
Gh_A07G2125-BD-F	ATGGAGGCCGAATTCATGAA CATGAATTATTTCC
Gh_A07G2125-BD-R	CAGGTCGACGGATCCCAAAAGCCACACATAC
Gh_A10G0207-BD-F	ATGGAGGCCGAATTCATGGAACCATCGGAGCTG
Gh_A10G0207-BD-R	CAGGTCGACGGATCCGAGAGTAAGAACAGATG
Gh_D08G0763-BD-F	ATGGAGGCCGAATTC ATGCATAAGAAAATGGC
Gh_D08G0763-BD-R	CAGGTCGACGGATCCGAAAGCCTCAATCCAGA
Gh_D10G0187-BD-F	ATGGAGGCCGAATTCATGGAACCATCGGAGCTG
Gh_D10G0187-BD-R	CAGGTCGACGGATCCGAGGGTAAGAACAGATGG
Gh_D11G0671-BD-F	ATGGAGGCCGAATTCATGAATTATTTCCCAGATGAAG
Gh_D11G0671-BD-R	CAGGTCGACGGATCCCAAAATCAACACATATTCTGG
GhUBQ7-F	AGGCATTCCACCTGACCAAC-
GhUBQ7-R	CAGCGAGCTTGACCTTCTTC
Gh_D08G0763-vigs-F	GTGAGTAAGGTTACCGAATTCTCAAGCTTGTAAGCC
Gh_D08G0763-vigs-R	CGTGAGCTCGGTACCGGATCCGCAAGTCCTTGCAAC
GhCLA1-vigs-F	CGACGACAAGACCGTGACCATGCACAACATCGATGATTTAG
GhCLA1-vigs-F	GAGGAGAAGAGCCGTCATTAGCATGAATGATGAGTAGATTGCAC
Gh_A07G2125-qPCR-F	CAGGTTTCTTAGGGAGCTG
Gh_A07G2125-qPCR-R	CCACACGGTTTAACCTCAA
Gh_A08G0662-qPCR-F	CCAAGGATGGTGATCAGACG
Gh_A08G0662-qPCR-R	GAGACCATCAGTTGAAAACC
Gh_A08G1014-qPCR-F	TTCGTGGGGAACTGTTACG
Gh_A08G1014-qPCR-R	CACTTGCAAATTCGTAAACG
Gh_A10G0207-qPCR-F	CATGCACTAAACTTCAGAGGC
Gh_A10G0207-qPCR-R	GAACGGGCAGTCTCGTATCTC
Gh_A11G1077-qPCR-F	GGAACTGTTACGCTGTTAGC
Gh_A11G1077-qPCR-R	TCCGCTTAAGTCGAATCTCC
Gh_D08G0763-qPCR-F	AACGCCTGGTGGGTAGATGT
Gh_D08G0763-qPCR-R	ACCACATCCCAAAATCCAGA
Gh_D08G1288-qPCR-F	GGACTCGAAGTGCTCGCATC
Gh_D08G1288-qPCR-R	ACAACCCGAAGAAACAGCGA
Gh_D11G1288-qPCR-F	GGACAAATACTTCGACCTGG
Gh_D11G1288-qPCR-R	CAACTTGGAAGCAGCATTCG

## Data Availability

The data presented in this study are available in this article.

## Supplementary Materials

The following supporting information can be downloaded at: https://www.mdpi.com/article/10.3390/plants13081152/s1, Figure S1. Expression profiles of GhTIR1/AFBs under cold, hot, salt and PEG. Figure S2. Yeast two-hybrid bait protein self-activation assay.
