# Supplementary figures and images for: Genome-Wide Analysis Elucidates the Roles of GhTIR1/AFB Genes Reveals the Function of Gh_D08G0763 (GhTIR1) in Cold Stress in G. hirsutum
Source: Plants (Basel). 2024 Apr 20;13(8):1152. doi: 10.3390/plants13081152 (PMC11055017; doi:10.3390/plants13081152)

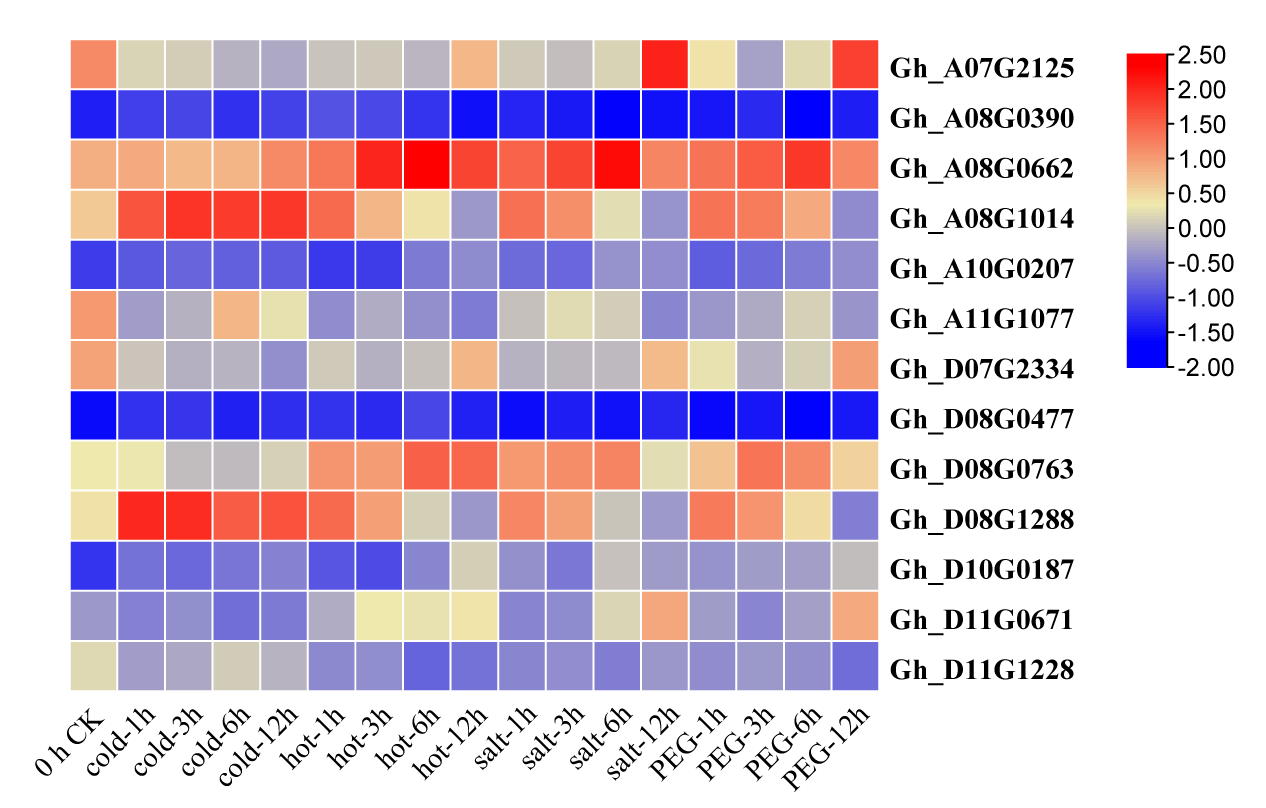

Supplement: Supplementary file 1 [file plants-13-01152-s001.zip › FigureS1.tif]

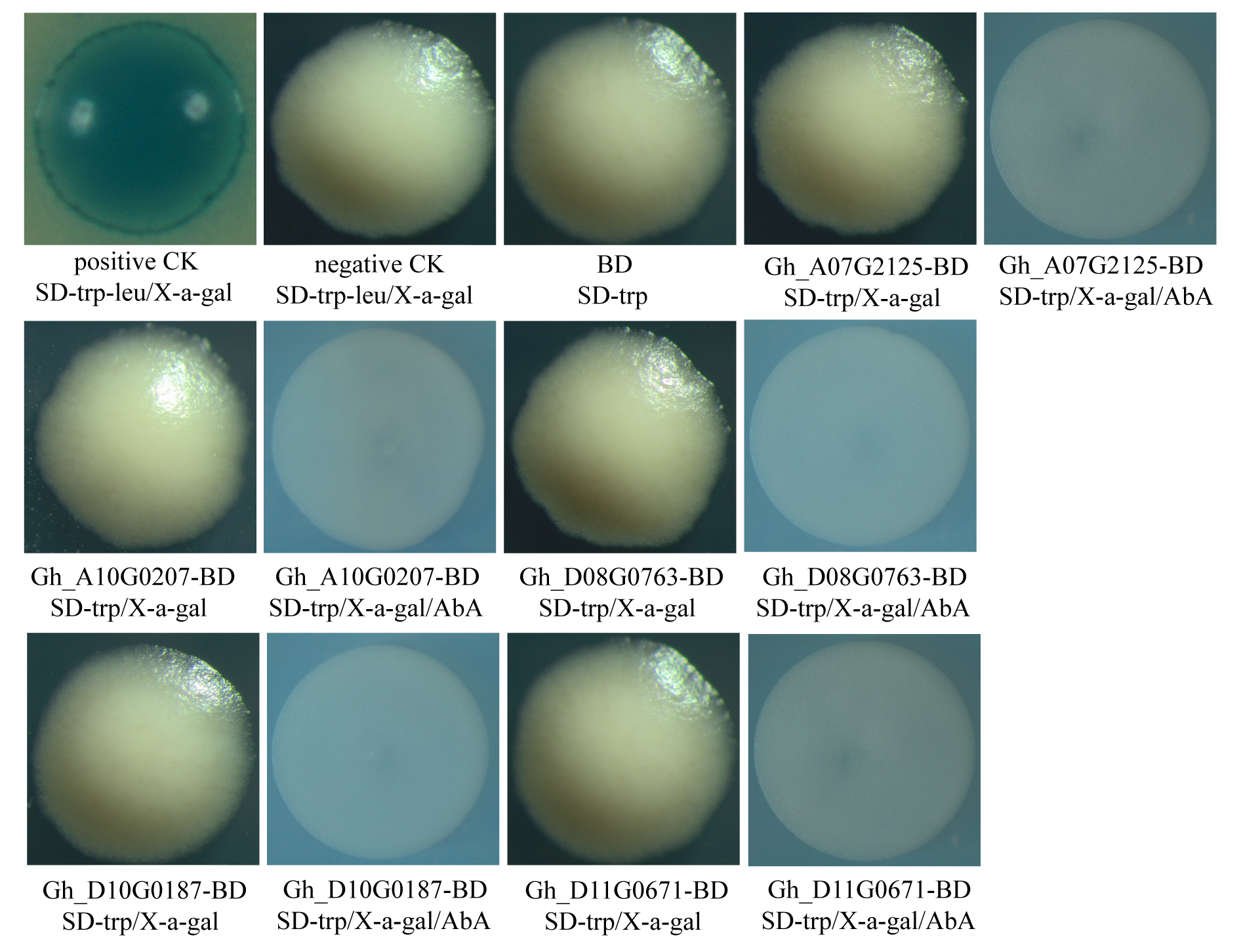

Supplement: Supplementary file 1 [file plants-13-01152-s001.zip › FigureS2.tif]
